# Coagulants: A Further Reply to Dr. Kirk

**Published:** 1894-11

**Authors:** Max Greenbaum

**Affiliations:** Philadelphia


### COAGULANTS: A FURTHER REPLY TO DR. KIRK.

BY MAX GREENBAUM, D.D.S., PHILADELPHIA.

THE position taken by Dr. Kirk, in his recent article "Coagulants in the Treatment of the Pulp-Chamber and Canals," has, we believe, been shown to be far from convincing, and far from its purpose to disprove the assertion that such coagulants as carbolic acid or zinc chloride are detrimental when employed in canal work. The main-stay of the argument appears to be the principle of osmotic action, and we may permit ourselves to say that if osmosis has anything to do with the determination as to whether certain coagulants should be excluded or included in the treatment of the root-canal, Dr. Kirk has not made it apparent that it is so. It seems reasonable that if it is desired to present an argument which should convince those whom he is criticising, no irrelevancy or deficiency should exist; and if they did exist, naturally a serious objection arises, and, aside from the inference which may be drawn that his opinion of his adversaries is altogether too small, otherwise his argument in its present shape would not have been imposed upon them. We may find a second objection, perhaps more serious than the first. This applies to knowledge concerning the action of coagulants. Dr. Kirk makes no distinction in the action of coagulants, and yet there is a very broad one. Some act in such manner that egg albumen is thoroughly cooked when placed in contact with them; others again but partially. If campho-phénique, or oil of cloves, or oil of cinnamon be brought in contact with egg albumen, we can observe readily that the resultant coagulum is quite different from the one produced with carbolic acid, or zinc chloride deliquesced, or corrosive sublimate used in such strength as will manifest its powerful coagulating quality.

Carbolic acid with egg albumen produces coagulation that is marked. In consistency it resembles the albuminoid part of a hard-boiled egg, being quite as hard and unyielding to touch. Visual test detects very little difference. With campho-phénique and egg albumen, on the other hand, we recognize distinguishing points at once. The coagulum is not as hard nor as unyielding, and compares more with the albuminoid portion of a soft-boiled egg. It presents a soggy appearance in contrast to the coagulum produced with carbolic acid, which appears much more solid. In short, we can say at once, albumen is not cooked as thoroughly in the one case as it is in the other.

Chloride of zinc deliquesced renders egg albumen almost as hard as cartilage. To separate a portion from its mass requires a greater force than that which would be sufficient to detach a portion were we dealing with coagulation from carbolic acid and albumen, while such coagula as result from applications of campho-phénique to albumen of eggs separate into smaller particles readily.

Blows from a smooth instrument leave no impression upon coagula from zinc chloride or carbolic acid, while in the same test with campho-phénique the opposite is the result. Where we impress an instrument so as to produce an indentation in the coagulated mass, in the first case the mass is so resisting that it resumes its original form upon removal of the instrument. These characteristics indicate a thorough cooking process as the result of deliquesced zinc chloride applications to egg albumen.

In contrast to these results we have those where the coagula are produced from the action of oil of cloves or oil of cinnamon upon egg albumen. These coagulants produce very little of the cooking process. The coagulations are flaky throughout, showing less consistency than even those from campho-phénique.

These observations may be confirmed by means of the microscope. Coagulations produced with campho-phénique, oil of cloves, or oil of cinnamon show numerous interstices throughout their mass. Coagulations from the action of carbolic acid discloses very little of any structure, while in such cases where zinc chloride had acted upon albumen, the resultant coagula show still less structure, appearing as a solid mass.

From this we may assume that there is a decided difference in the action of coagulants. But so far this difference exerts little influence. Not until we observe its effect in the treatment of the root-canal, with its possible and probable sequences, do we ascertain that Dr. Kirk, with his principle of osmotic action, is far from the safe point in the treatment of devitalized teeth. The position taken by him leaves the interpretation that diffusion of liquids takes place in the root-canal even after the use of such coagulants as carbolic acid or zinc chloride, and thus any after-application that may be directed to such canal continues to act upon the principle of diffusion, so that the objection extended by many that the use of coagulants (meaning irritating coagulants) in the canal renders a barrier to any further diffusion is shown to be erroneous. Now, we think we may readily observe that Dr. Kirk's argument is faulty. We found different coagulants act in different degrees as relates to power of coagulation,—the different coagula showing a marked difference in structure. Some were found to be loosely coagulated, others solidly coagulated; and we may find, furthermore, that albumen coagulated solidly, such as results from the action of carbolic acid or zinc chloride, admits of no extented penetration of a second application, unless such application remained in contact in excessive quantity with the coagulum for two days or more. In dental work this is not admissible. Not alone that circumstances very frequently will not permit the excessive quantity being placed in apposition with the root-structure; but, supposing they did, it would be inconvenient and annoying to do so. When to this we add the grave objection, pointed out elsewhere, that under no circumstances could we regulate just sufficient diffusion and coagulation that it would not be liable to irritate the pericemental membrane, we may find that Dr. Kirk not alone has failed to impeach the position of those who do not employ irritating coagulants, such as carbolic acid and zinc chloride in the treatment of the root-canal, but he has called forth fresh objections to the use of such medicaments.

Coagula produced by the action of campho-phénique, oil of cloves, oil of cinnamon, or any coagulants that produce with albuminoid matter simply such coagulations as would present the characteristics of loosely-formed coagula, admit diffusion of a second application that stands in decided contrast to that of hard-formed coagulations. They are of such nature that second applications penetrate throughout their respective masses almost instantly.

Now, from these observations we may conclude, whereas the action of carbolic acid, zinc chloride, or any coagulants which produce with albuminoid matter coagulation, the nature of which renders diffusion of a second application difficult, or retards it to such extent that in dental work we would have little, if any penetration, that such practice is unscientific, to say the least, because of the untoward results that may ensue. And, moreover, as the use of such coagulants as campho-phénique, oil of cloves, oil of cinnamon, or any coagulants the use of which with albuminoid matter renders the resultant coagula easy to diffusion of any subsequent application, such treatment, to the contrary, is scientific.

There is no need to enter again into the details of the objections that beset Dr. Kirk's argument. The principle of osmotic action in its entire understanding, instead of offering a scientific basis for the use of certain coagulants in canal work, offers a scientific basis against such practice. Its acceptance commits us to alternate difficulties. And, in conclusion, we may add that it seems curious in what manner certain pathological teachings that have been in vogue for the past twenty-five years, in the light of advanced knowledge, assume a scientific aspect heretofore denied them. It has been the accepted procedure with many for years to exclude irritating coagulants from the treatment of devitalized teeth, observation and experience furnishing the basis for the restriction; and to-day these teachings become verified from actual experimentation. Then, again, there is a peculiar coincidence in the fact that the pioneers in this work have been identified with the work that has almost completely revolutionized operative dentistry from the stand-point of filling cavities of decay, giving rise to the *New School.* That work, too, owes its inception to empiricism. But to-day science underlies empiricism. The statements of laws of any scientific knowledge are the statements of laws of scientific dentistry, and thus it advances to the position of a true science.

